# Patient attitudes towards community-based tuberculosis DOT and adherence to treatment in an urban setting; Kampala, Uganda

**DOI:** 10.11604/pamj.2017.27.1.11119

**Published:** 2017-05-01

**Authors:** Sempeera Hassard, Anguzu Ronald, Kawooya Angella

**Affiliations:** 1 School of Public Health, Makerere University, Kampala, Uganda; 2 International Health Sciences University

**Keywords:** Tuberculosis, pulmonary tuberculosis, tuberculosis directly observed treatment, community-based, treatment supporters

## Abstract

**Introduction:**

High Tuberculosis treatment default rate (17%) and sub-optimal treatment completion rates (45%) has burdened Kampala. Nevertheless, there are observable increase in the number of patients on TB DOT; from 6% to 29% in two consecutive annual reports. The main objective was to determine the association of TB patient attitudes towards community-based observers on the TB drug adherence on directly observed treatment for TB in Kampala.

**Methods:**

A cross-sectional study was carried out in Lubaga division, Kampala. A total of 201 patients in continuation phase of treatment for Pulmonary TB (i.e. 8 to 20 weeks of TB treatment) were included in the study. Patient attitudes were measured using a 4-point Likert scale aggregated into a binary outcome with ''agree'' and ''disagree'' responses. Poisson regression model using a forward fitting approach in STATA v12 was used to determine the association between patient attitude towards CB-DOTs observers and adherence to TB treatment.

**Results:**

Among the 201 patients, 66% reported their treatment was being observed by someone. Relatives were the commonest (82%) treatment observers, 26% were non adherent to their TB treatment. Perceiving ''no need for a treatment observer'' and ''people rejecting TB patients'' were predictors of non-adherence to TB treatment (IRR=1.6,95%CI 1.00-2.57;p=0.048) and (IRR=0.6, 95%CI 0.35-0.95; p=0.019) respectively.

**Conclusion:**

Patient's perceived attitude and stigma towards treatment observers contribute to non-adherence on TB treatment. For improved local TB control, more emphasis is needed to build a friendly environment between treatment supporters and patients during the course of TB treatment.

## Introduction

Tuberculosis (TB) remains a major public health concern in many developing countries [1], despite the TB incidence falling globally for several years; 2% per year in 2012 [2]. It is estimated that over, 8.6 million new TB cases and 1.3million TB deaths occurred in 2012 [3]. Sub-Saharan Africa carries the greatest proportion of new cases per population; over 260 cases per 100 000 population in 2011 showing a reduction compared to the incidence by the year 2010; 271 case per 100 000 population. Nevertheless, mortality rate due to TB has reduced by 45% since 1990 and TB incidence rates are falling in most parts of the world [4]. This major success has been attributed to the international rollout of the DOTS strategy for about 18 years back [5]. Uganda is ranked 16th among the 22 TB high burden countries in the world with 136 new smear positive cases per 100,000 people per year [6]. The high TB burden has been attributed to the high prevalence of HIV/AIDS, where over 60% of Ugandan TB patients have HIV/AIDS [7]. After its pilot in 1998 and scale up in 2005, directly observed treatment strategy (DOTS) has produced remarkable improvement in the control of TB in Uganda; marked by drastic increase in the treatment success rate among smear positive TB patients of 13% since 1998 (62% in 1998to 75% in 2007) [8]. Over the years, patients on TB DOT have increased from 47% to 55% for the 2011 and 2014 respectively. Kampala city holds about 5% of Uganda's population with a population growth rate of 4.1%; higher than the national average. This has led to the rapid multiplication of many slum communities in the city which now accounts for over 60% of the city population. These slums are pockets of high HIV prevalence (9.3%), the leading drivers of the TB burden. It's estimated that Kampala city contributes about 20% Uganda's TB burden despite the recent increase in number of patients on TB DOTS [9, 10]. In 2012, Kampala registered an overwhelming increase in the number of patients on TB DOT; from 6% to 29% for the April - June and July -Sept 2012 quarter reports respectively. However, this striking achievement seems not to reflect the relatively high defaulting on TB treatment of 17% and the low treatment completion rates of only 45% [11]. This discrepancy raises questions about the implementation of CB-TB DOT in this division that need immediate answers. Therefore, this study seeks to assess the association between patients? perceived attitude and stigma towards community-based TB DOT on adherence to Tuberculosis treatment in Kampala, central Uganda.

## Methods

**Study setting, design and population**: A cross-sectional study was carried out in Lubaga one of the administrative divisions of Kampala the capital city of Uganda from August 2014 to October 2014. This division was purposively selected because it registered the highest number of patients on TB DOT (70%) for the quarterly report of Jan to March for the 2013/14 financial year among the divisions of Kampala. Specifically data was collected from all health facilities in Lubaga Division that included; Kawala and Kitebi. Kisenyi health centre is not part of Lubaga division but was included in the study due to its close proximity to Lubaga division that many patients from this division seek their health services from this health facility. Patients on TB treatment participated in the study.

**Inclusion criteria and exclusion criteria**: Patients diagnosed with pulmonary TB and have been on TB treatment for at least 8 weeks but not more than 20 weeks were eligible for the study. This category was chosen because it could enable us get the estimated sample size given the short time for data collection we had. Patients who were weak or incapacitated were excluded from the study. In addition, patients on re-treatment were not included in the study due to the likelihood of behavior change during the second time of treatment. Patients regarded as transfer in to these facilities were also not included in the study because these were likely to be different from those who started their treatment at the three health facilities.

**Sample size determination and Selection of patients**: Kish Leslie formula was used to determine the sample size. Using a modified Leslie Kish formula (1965) [12], p=69% (proportion of patients reported to be under community-based TB DOT by September 2012) (KCCA, quarterly report 2014), precision, e=0.05 and after adjusting for the total number of patients on TB treatment by June 2014 in Lubaga division , a sample size of 201 was computed. Selection of TB patients was conducted using systematic sampling. In-charges of the TB clinics were requested to give an average number of patients that attend the clinic on a typical clinic day. This total was divided by the sample size from which the sampling interval was calculated. On respondents exit from the TB clinic, patients were consecutively counted and every fifth person was selected for interviews. If the patient selected was not eligible or declined to participate in the study, re-counting was done from the next TB patient exiting the TB clinic until the estimated sample size was achieved.

**Study measurement**: The outcome variable was patient adherence to TB treatment. This was measured as binary variable, that is to say, being adherent or not. In the last 4 weeks, patients were categorized as being adherent on TB treatment if they reported taking at least 90% of their doses, whereas non adherent was defined as those who took less than 90% of their doses [13]. Attitude was measured using statements that respondent had to answer on a 4 point Likert scale (agree, strongly agree, disagree and strongly disagree). Other independent variables included; Socio-demographic characteristics and attitudes towards TB DOT observers.

**Analysis**: Descriptive analysis was done for all variables and reported using percentages. Means and their standard deviations (SD) were calculated for continuous data variables. At bivariate analysis, fisher's exact test run to compute significant association between the adherent and non-adherent TB patients; p≤0.05 was statistically significant. Key variables cross-tabulated with TB drug adherence were patient's socio-demographic characteristics, utilization of TB DOT services and attitude towards TB DOT observers. For TB patient attitudes, responses from the 4 point Likert scale were aggregated into ''agree'' and ''disagree'' in order to generate a binary categorical outcome of their responses. At multi-variable analysis, the association of patient's attitude towards community-based treatment observers and adherence to TB treatment was determined using Poisson regression model. The incidence risk ratios (IRR) at 95% confidence interval and robust standard errors were estimated. The Poisson regression model was used because the outcome variable was greater than 10%. All variables in the bivariate analysis with p≤0.05 or potential confounders were included in the multi-variable analysis using forward approach. All statistical analyses were done using STATA version 12.

**Ethical consideration**: The study obtained ethical approval from the International Health Sciences University (IHSU) student research review board. Detailed patient information was given explaining the purpose of the study, confidentiality and privacy of their information emphasized. Informed consent from all participants was obtained before enrollment to participate in the study. Permission was obtained from the Kampala Capital City authority (KCCA) and the in-charges of health facilities to carry out research in their TB clinics.

## Results

**Socio-demographic characteristics of study participants**: A total of 201 patients participated in the study. Of these, 66% were on continuation phase of treatment. The mean age of respondents was 30 years (SD±7) and more than half (58%) of the participants were males, single (never married, widowed or divorced). The most common level of education was secondary (45%) and majority (34%) of the respondents were not earning at all by the interview date. Females were more adherent to treatment than males (31% versus 69%; p=0.245) whereas patients on the initial phase were more adherent than those on continuation (37% versus 63%; p=0.344) Table 1.

**Table 1:** Socio-demographic characteristics of study participants

Variable	Overall n*(%)	Adherent on TB treatment		*p*-valueⱡ
		Yes n (%)	No n (%)	
**Sex**				
Male	116 (58)	89 (77)	27 (23)	0.245
Female	85 (42)	59 (69)	26 (31)	
**Ageɫ**				
18-24	42 (21)	30 (71)	12 (29)	0.525
25-34	57 (28)	57 (79)	15 (21)	
35-44	38 (19)	33 (67)	16 (33)	
45-66	64 (32)	28 (74)	10 (26)	
**Marital status**				
Single	143 (71)	104 (73)	39 (27)	0.648
Married	58 (29)	44 (76)	14 (24)	
**Education level**				
None	17 (8)	11 (65)	6 (35)	0.524
Primary	73 (36)	51 (70)	22 (30)	
Secondary	90 (45)	69 (77)	21 (23)	
Tertiary/university	21(11)	17 (81)	4 (19)	
**Income ᶴ**				
None	71 (35)	54 (76)	17 (24)	0.830
<50,000	56 (28)	40 (71)	16 (29)	
>50,000	74 (37)	54 (73)	20 (27)	
**Duration on TB Treatment (Phase)**				
Initial	69 (34)	48 (70)	21 (37)	0.344
Continuation	132 (66)	100 (76)	32 (24)	

ɫ Mean age (SD): 30 years (7)

ⱡ Fishers exact test

ᶴ Ugandan shillings (1 USD=3370 UGX) Bank of Uganda (14)

**Utilization of community-based TB DOT services and adherence to TB treatment**: Two thirds (66%) of TB patients had their TB treatment observed by a treatment supporter. Majority (61%) of the treatment observers were relatives or friends. More than a third (35%) of patients had taken TB medication without their treatment observers. The most common reason for not having someone to observe TB treatment was living alone or lacking a household member (59%). Almost equal proportions of patients having and not having treatment observers were not adherent on TB treatment respectively (26% versus 28%: p=0786). Two fifths of participants (36%) of patients whose reason for not having a treatment observer was ''do not want'' were non-adherent on TB treatment. Three out of ten (26%) of all TB patients were not adherent to TB treatment Table 2.

**Table 2:** Utilization of Community-based DOT services for anti-TB treatment

Characteristic	Overall (n*)%	Adherent on TB treatment		*p*-value
		Yes n (%)	No n (%)
**Do you have someone observing your treatment**				
No	69 (34)	50 (72)	19 (28)	0.786
Yes	132 (66)	98 (74)	34 (26)	
**How are you related to this person**				
No treatment supporter/observer	69 (34)	50 (72)	19 (28)	0.879
Relative/Friend	122 (61)	90 (74)	32 (26)	
Designated CB-DOTs observer	10 (5)	8 (80)	2 (20)	
**Have you ever taken medication without a supporter? (n=132)**				
No	62 (47)	48 (77)	19 (23)	0.552
Yes	70 (53)	50 (71)	14 (29)	
**Why did you take medication without this person (n=70)**				
Patient away from home	32 (75)	24 (34)	8 (66)	0.544
Treatment observer away	38 (54)	26 (68)	12 (32)	
**Reasons for not having Treatment observer (n=69)**				
Staying alone	41 (59)	32 (78)	9 (22)	0.275
Don’t want/can take medication alone	28 (41)	18 (64)	10 (36)	

**Perceived Attitude and stigma towards community-based TB DOTs**: Three quarters of patients 149 (74%) like the TB DOT idea; of these majority 115 (77%) were adherent to treatment (p=0.067). More than a third (34%) of patients felt there was no need for a treatment observer of whom 24 (35%) were not adherent to TB treatment (p=0.064). A total of 125 (62%) of patients disagreed to a statement ''having treatment observers is time consuming to the patient'', however there was no significant difference between patients non adherent to treatment; 26% and 28% respectively. Seventy eight (39%) of patients did not want their TB status to be known to another person and out of these, 23 (29%) were non adherent to TB treatment. Up to134 (67%) of patients agreed that treatment observers would expose their TB status but a lesser proportion was non adherent to treatment than those who disagreed; 24% and 31% respectively (p=0.309). Majority of patients agreed that people reject TB patients 121(60%) and among them, 1 in 5 was non adherent to TB treatment Table 3.

**Table 3:** Perceived Attitude towards treatment observers

Characteristic	Overall	Adherent on TB treatment		*p*-value
	(n)%	Yes n (%)	No n (%)	
**I do not need a treatment observer**				
Disagree	132 (66)	103 (78)	29 (22)	0.064
Agree	69 (34)	45 (65)	24 (35)	
**No observer has time for a TB patient**				
Disagree	129 (64)	96 (74)	33(26)	0.741
Agree	72 (36)	52 (72)	20 (28)	
**I would like the TB DOT idea**				
Disagree	52 (26)	33 (63)	19 (37)	0.067
Agree	149 (74)	115 (77)	34 (23)	
**Having treatment observer is time consuming to the patient**				
Disagree	125 (62)	93 (74)	32 (26)	0.745
Agree	76 (38)	55 (72)	21 (28)	
**Don’t want any other person to know I have TB**				
Disagree	123 (61)	93 (76)	30 (24)	0.511
Agree	78 (39)	55 (71)	23 (29)	
**Observers will expose my TB status**				
Disagree	67 (33)	46 (69)	21 (31)	0.309
Agree	134 (67)	102 (76)	32 (24)	
**People reject TB patients**				
Disagree	80 (40)	53 (66)	27 (34)	0.072
Agree	121 (60)	95 (79)	26 (21)	

**Multivariate analysis of the factors affecting the implementation of community-based TB DOTS**: At multivariate analysis, agreeing to ''I do not need a treatment observer'' and agreeing ''to people reject TB patients'' were significantly associated to non-adherence to treatment; IRR=1.6, CI (1.00-2.57; p=0.048) and IRR=0.6, CI (0.35-0.95; p=0.019) respectively Table 4.

**Table 4:** Multivariate analysis of the factors affecting the implementation of community-based TB DOTS

Variable	Unadjusted IRR(CI)	Adjusted IRR (CI)	p-value
**I do not need a treatment observer**			
Disagree	1	**1**	
Agree	1.58 (1.00-2.50)	1.60 (1.00-2.57)**	**0.048**
**I would like the TB DOT treatment**			
Disagree	1	**1**	
Agree	0.62 (0.39-0.99)	**0.7 (0.43-1.14)**	0.152
**People reject TB patients**			
Disagree	1	**1**	
Agree	0.63 (0.40-1.00)	**0.37 (0.37-0.92)**	**0.019**

## Discussion

The objective of this study was to determine the association between patients' attitude and stigma towards community-based TB DOT and adherence to TB treatment. Our findings suggested that patients who expressed no need for a treatment observer were significantly associated with non-adherence to TB treatment. Our finding was also revealed as a risk factor associated with defaulting from TB treatment in case-control study in South Africa [14]. In Uganda, stigma towards TB is still high; many patients would wish to have their TB status confidential due to the misconception that whoever has TB also has HIV. This was evident from our results as two out of five (39%) of TB patients did not want any other person to know their TB status. It's an indication that some patients still see themselves ashamed of getting TB [15]; such social pressures resulting in anxiety over community perceptions of one having TB [16] affect patients confidence in the community resulting in non-adherence to treatment [17]. This therefore calls for more counseling to patients diagnosed with TB. Counseling would increase patient's confidence and hope after TB treatment thus improving their adherence to treatment. The results also indicated that a feeling of rejection by the community was significantly associated with non-adherence to TB treatment. This is consistent with studies that have looked at community involvement in the community based, directly observed TB treatment. This is an indication of the high stigma towards TB. Stigma has been noted as one of the most important risk factor for non- adherence [18–20]. During treatment TB patients require a lot of support, but this is not the case in some communities [21]; instead, patients are isolated because communities fear that they will contract the disease. This in the end affects the patient as they would fear to be seen taking their medication regularly. Therefore this calls for continuing sensitization of communities so as to increase the knowledge base about TB.

**Study limitations**: The analysis purely depended on only quantitative data and lacked qualitative data to supplement our understanding of different aspects of patients' perceived attitude and stigma towards CB-TB DOT observers. In addition, the study did not use standard question to measure attitude in this aspect; however, we think this can spark a wider understanding to the CB-TB DOT intervention program on the sides of patients to better local and global TB control. This was a cross-sectional study and therefore casual temporality cannot be determined.

## Conclusion

Patient's perceived attitude and stigma towards treatment observers may be a contributing factor affecting adherence on TB treatment. TB patient perspectives should be considered during CB-DOTS strategy reviews because their perceptions and stigma towards them may be enablers or barriers to achieving TB control goals. Therefore, emphasis is need to building a friendly environment between treatment supporters and patients during the course of TB treatment.

### What is known about this topic

- Tuberculosis is the leading cause of death among people living with HIV/AIDS. Sub-Saharan Africa has nearly all high TB burden countries disproportionately contributing highest TB related mortality globally;
- Well implemented community based TB Directly Observed Treatment (CB-DOTS) is known to improve treatment success rates;
- Patient's attitude towards healthcare services has been consistently found to affect the health service utilization.

### What this study adds

- Gives an insight into how community-based TB DOT is implemented among ethnically diverse and dynamic populations in an urban setting of a developing country;
- This study provides new knowledge to the global researchers on how attitudes of urban dwelling TB patient's would affect their utilization of community-based TB DOT;
- Our study identified community perception, attitudes and stigma associated with TB treatment and potentially treatment success and outcomes. This provides programmatic opportunity to improve quality DOTS expansion and enhancement.

## Competing interests

The authors declare no competing interests.
